# Children’s Non-symbolic, Symbolic Addition and Their Mapping Capacity at 4–7 Years Old

**DOI:** 10.3389/fpsyg.2017.01203

**Published:** 2017-07-17

**Authors:** Yanjun Li, Meng Zhang, Yinghe Chen, Xiaoshuang Zhu, Zhijun Deng, Shijia Yan

**Affiliations:** ^1^Institute of Developmental Psychology, Faculty of Psychology, Bejing Normal University Beijing, China; ^2^Department of Psychology, Rutgers, The State University of New Jersey, New Brunswick NJ, United States; ^3^Institute of Information Control, China Aerospace Academy of Systems Science and Engineering Beijing, China

**Keywords:** non-symbolic addition, symbolic addition, developmental trajectories, mapping, children

## Abstract

The study aimed to examine the developmental trajectories of non-symbolic and symbolic addition capacities in children and the mapping ability between these two. We assessed 106 4- to 7-year-old children and found that 4-year-olds were able to do non-symbolic addition but not symbolic addition. Five-year-olds and older were able to do symbolic addition and their performance in symbolic addition exceeded non-symbolic addition in grade 1 (approximate age 7). These results suggested non-symbolic addition ability emerges earlier and is less affected by formal mathematical education than symbolic addition. Meanwhile, we tested children’s bi-directional mapping ability using a novel task and found that children were able to map between symbolic and non-symbolic representations of number at age 5. Their ability in mapping non-symbolic to symbolic number became more proficient in grade 1 (approximate age 7). This suggests children at age 7 have developed a relatively mature symbolic representation system.

## Introduction

### The Developmental Trajectories of Non-symbolic and Symbolic Addition Capacities

The capacity of non-symbolic addition is a skill of quantity calculation, which is based on the non-symbolic representation system, such as dots. It is widely agreed that the non-symbolic addition ability was based on the approximate number system (ANS) system (Barth et al., 2005, 2006, 2008). This system has three features. First, it is an inherent and universal system shared by animals and humans (Wynn, 1992; Flombaum et al., 2005). It is not affected by culture (Pica et al., 2004). Second, it represents quantities in an approximate way (Feigenson et al., 2004). Third, the precision of ANS system increases with age (Halberda et al., 2008). However, ANS system is not sufficient for doing symbolic addition. The capacity of symbolic addition also relies on the symbolic representation system to calculate quantities. The symbolic representation system is different from the ANS system in three ways. First, it is an acquired system, which is affected by the language faculties (Pica et al., 2004; Xenidou-Dervou et al., 2015). Second, the system represents quantities precisely (Izard and Dehaene, 2008; Mussolin et al., 2014). Third, the range of number and the accuracy that this system is able to manipulate increase with age (Halberda et al., 2008; Praet and Desoete, 2014).

Barth et al. (2005, 2006, 2008) demonstrated 5-year-old children who have not yet received any formal math instructions could complete the non-symbolic addition task with different types of stimulus. The non-symbolic arithmetic ability has been attributed to the so-called approximate number system (ANS) (Feigenson et al., 2004; Barth et al., 2005, 2006, 2008; Dehaene, 2011). ANS representations were claimed to be available innately and support non-symbolic arithmetic (McCrink and Wynn, 2004, 2009). Still, it is not clear whether children as young as 4 years are able to complete non-symbolic addition tasks.

With increasing age, children develop higher-order mathematical abilities, which are based on the symbolic representation system, in other words, on the use of symbols, such as Arabic numbers, for representing quantities. Researchers proposed that the development of symbolic arithmetic ability was related to culture and formal education; children’s symbolic addition skills develop rapidly after entering primary school (Geary, 2000; Baroody and Dowker, 2003). However, Gilmore et al’s (2007) study showed that children were able to do symbolic addition tasks with large numerosities at the age of 5, before formal mathematical education. They argued that children might solve the task with the help of the ANS. It is plausible that they converted symbolic Arabic numbers to non-symbolic numerosities and then added these numerosities. Moreover, although children at 5 years old have not obtained mathematical education from school, they may have already been exposed to many informal mathematic activities, such as playing number board game, reading stories involved quantities, and so on. Such informal math activities help improve children’s symbolic skills (Kleemans et al., 2012; Skwarchuk et al., 2014; Berkowitz et al., 2015; Benavides-Varela et al., 2016). Up to now, the empirical evidence for the onset of symbolic addition capacity in children has not reached a consensus. This study considered both symbolic and non-symbolic addition capacities and aimed to further explore this issue.

Toll et al. (2015) investigated children’s non-symbolic and symbolic comparison skills and found children became proficient in the symbolic representation system by the first grade. These results indicate although children’s non-symbolic ability develops continuously over time (Halberda et al., 2008), their symbolic ability develops remarkably with the start of formal schooling. Xenidou-Dervou et al. (2015) assessed children’s non-symbolic and symbolic addition capacities in kindergarten and at the beginning of primary school. They found 5-year-old children were already able to perform non-symbolic addition and that they showed a ratio effect. In contrast, children’s symbolic addition capacity began in grade 1 (approximately 6 years old), at the start of formal schooling but not earlier. According to the authors, children’s increasing linguistic proficiency, such as their number naming ability, could affect the development of their ability for symbolic addition. In contrast, the development of non-symbolic addition should not be affected by language (Praet and Desoete, 2014; Xenidou-Dervou et al., 2015). Therefore, Xenidou-Dervou et al. (2015) claimed that the non-symbolic and symbolic addition relies on two distinct systems. However, the ratio effect that Xenidou-Dervou et al. (2015) found does not fully reflect children’s competence in symbolic or non-symbolic addition. Other researchers (Gilmore et al., 2007; Chen and Li, 2014; van Marle et al., 2014) believed both non-symbolic and symbolic addition abilities, in some extent, rely on the ANS system. It is known that ANS system had an important influence on non-symbolic addition ability (McCrink and Wynn, 2004, 2009; Barth et al., 2005, 2006, 2008). Chen and Li’s (2014) meta-analysis indicated the non-symbolic comparison acuity had a significantly positive correlation with mathematical performance. However, Fazio et al. (2014) showed that the relationship between non-symbolic numerical magnitude knowledge and mathematical achievement was relatively weak than the relationship between symbolic numerical magnitude knowledge and mathematical achievement, especially for children 6 years old and older. The available evidence displayed both distinctions and connections between symbolic and non-symbolic addition abilities, but the development trajectories of these two and their relationships with the ANS are still unclear. The present study aims to provide more comprehensive developmental trajectories of non-symbolic and symbolic addition capacities in preschoolers and young primary students.

### The Development of Mapping Ability

When children face with tasks including both non-symbolic and symbolic information, they need to map one type of information to the other, or vice versa. The ability of building correspondence between the symbolic and non-symbolic numerical information is called the mapping capacity (Mundy and Gilmore, 2009). It is an important ability that was showed to predict children’s mathematics achievement in schools (Mundy and Gilmore, 2009; Brankaer et al., 2014; Praet and Desoete, 2014; Friso-van Den Bos et al., 2015), but we currently know little about when and how this ability develops in children.

Some researchers (Gilmore et al., 2007; Holloway and Ansari, 2009) stated the numerical distance effect found in symbolic numerical tasks indicated that 5-year-old children mapped symbolic representations onto the preexisting non-symbolic representations of quantity. However, this interpretation is not convincing, because with only symbolic information presented in their tasks, it is possible that children did not have to map symbolic to non-symbolic information. To test children’s mapping ability more directly, Lipton and Spelke (2005) asked 5-year-old children to complete three tasks: (1) verbally estimate the number of items in a set, (2) choose one set out of two with a given number of items, and (3) estimate the number of items after letting them know how many items another items contained. They found that children were able to map number words onto non-symbolic magnitudes once they mastered counting skill. However, their free response tasks were difficult to 5-year-old children, many of them failed to produce estimation at all. Odic et al. (2015) tested 2- to 5-year-old children’s mapping ability between the ANS and number words. For the ANS-to-Word task, children were asked to guess the number of dots on the card. For the Word-to-ANS task, they asked children to pat a stuffed tiger for certain number of times (i.e., pat the tiger task). They found children were able to map number words to the ANS successfully before 4 years old, but their ability to map the ANS to number words appeared after age 4. Notably, in their pat-the-tiger task, although number words involve some kind of symbolic information, it is not delivered via the same modality as written Arabic numbers. Using various tasks, previous studies produced different, even inconsistent results. The current study aims to provide more valid empirically data on children’s development of mapping ability.

Mapping occurs in two directions, either from non-symbolic to symbolic representation or from symbolic to non-symbolic representation (Izard and Dehaene, 2008; Mundy and Gilmore, 2009). Mundy and Gilmore (2009) used novel tasks to measure 6- and 8-year-old children’s bi-directional mapping ability directly. Children were either presented with an Arabic symbol and asked to choose from two sets of dot arrays (i.e., mapping from symbolic to non-symbolic quantity) or presented with a set of dot arrays and were asked to choose from two Arabic symbols (i.e., mapping from non-symbolic to symbolic quantity). They found children’s mapping ability developed during 6–8 years old and they were more accurate on problems that involved mapping from non-symbolic to symbolic representation. They think this asymmetry in the two mapping directions stems from the precision of the two representations (Mundy and Gilmore, 2009). Izard and Dehaene (2008) proposed a model about mapping between symbolic and non-symbolic representations. In their model, symbolic information corresponds to precise points on the number line, whereas, non-symbolic representation is approximate. According to this model, Mundy and Gilmore’s (2009) task on mapping from symbolic to non-symbolic involves one precise point and two approximate regions on the number line; their task on mapping from non-symbolic to symbolic involves one approximate region and two precise points on the number line. Therefore, children’s higher accuracy in mapping non-symbolic to symbolic quantities, may because fewer approximate representations were involved. Brankaer et al. (2014) conducted a similar study as Mundy and Gilmore’s (2009) but they did not find the superiority in mapping non-symbolic to symbolic representation. If children are indeed more proficient in non-symbolic to symbolic mapping, to a certain extent, it implies children have a relative sufficient symbolic representation system to be mapped upon. Furthermore, this symbolic representation system is the basis for the development of advanced mathematical ability (Obersteiner et al., 2013; Linsen et al., 2015; Toll et al., 2015). Therefore, it is necessary to further examine whether or not children are more proficient in non-symbolic to symbolic mapping, and if so, when this advantage appears.

### Present Study

In sum, this study aims to achieve two goals. First, we aim to provide detailed development trajectories of non-symbolic and symbolic addition skills during the childhood. To our knowledge, previous research focused more on non-symbolic and symbolic addition skills in 5-year-olds and older (Barth et al., 2005, 2006, 2008). Gilmore et al. (2007) demonstrated that symbolic addition capacity onsets at the age of 5, before starting primary school instruction. However, Xenidou-Dervou et al.’s (2015) study showed symbolic addition onsets approximately 6 year old, with the start of formal schooling. How do we reconcile this conflict empirical evidence? What do the capacities of non-symbolic and symbolic addition look like in younger children, such as 4-year-olds? In this study, we assessed children’s symbolic addition capacities with the consideration of possible impact from language. Therefore, children’s verbal-counting, number-naming and symbolic ability were also tested. We predicted children’s symbolic addition ability would improve largely once they obtain the formal mathematical education, and it would exceed the non-symbolic ability at the start of primary school.

Second, this study aims to investigate when and how the mapping ability develops during childhood. With various tasks, previous research produced inconsistent results on children’s mapping ability (Lipton and Spelke, 2005; Holloway and Ansari, 2009; Mundy and Gilmore, 2009). To reconcile previous results and test mapping more directly, we created the hybrid representation addition tasks based on previous paradigms (Gilmore et al., 2007; Mundy and Gilmore, 2009). These tasks included both non-symbolic and symbolic representation numerosities, and they are designed to measure mapping abilities in both directions. Hybrid addition tasks are more difficult than previous tasks. Children have to map between the two systems and also add the two quantities at the same time. Therefore, successes in these tasks will strongly demonstrate children’s mapping ability. Based on previous findings (Gilmore et al., 2007; Mundy and Gilmore, 2009; Xenidou-Dervou et al., 2015), we hypothesized that children’s mapping ability appears at 5 years old and their mapping ability from non-symbolic to symbolic representation exceeds the other direction at 7 years old, when they obtain formal mathematical education.

## Materials and Methods

### Ethics Statement

This research was approved by the local ethical committee of Beijing Normal University. We obtained informed written consent from caretakers or guardians on behalf of the child participants involved in the study, according to the institutional guidelines of Beijing Normal University.

### Participants

A total of 106 children (59 girls) were recruited from three public schools located in Beijing, China. Two children were excluded due to their inability to complete the verbal-counting task (each with a 0 score), and other two children were removed due to their failure in the number-naming task (each with a 0 score). Twenty-eight 4-year-olds (*M* = 44.0 months, *SD* = 6.3), 30 5-year-olds (*M* = 60.8 months, *SD* = 3.0), and 24 6-year-olds (*M* = 72.5 months, *SD* = 3.9) recruited from two kindergartens and 20 7-years-olds (*M* = 83.2 months, *SD* = 2.5) recruited from the 1st grade of one primary school participated this study. All children were tested in the middle of the Chinese academic year (around December). All children are Mandarin native speakers. They were mostly from families of middle socioeconomic status. All children gave oral consent and their parents gave written consent before participation in the study. A gift (i.e., a notebook) was sent to each child for participation.

### Measures

#### Number-Naming

Children’s number-naming ability was measured. They were asked to read loudly 15 Arabic numbers, which were numbers included in the following tests. The numbers were written in three lines on one corkboard (36 cm × 12.5 cm). Numbers on the first line were 5, 6, 7, 8, 9, numbers on the second line were 11, 12, 14, 16, 18, and those on the third line were 21, 26, 27, 28, 32. Children obtained 1 point for successfully naming all numbers in one line. Otherwise, they obtained 0 point. Children who scored 0 were excluded, there for the total scores ranged from 1 to 3.

#### Verbal-Counting

Verbal-counting skills were assessed using the subtest from the Counting Sequence Knowledge Test (Zhang X. et al., 2014; Zhang J. et al., 2014). Children were asked to count loudly 5–11, 12–18, 19–24, 25–31, and 32–36. They obtained 1 point for successfully counting one full sequence. Otherwise, they obtained 0 point. Children who scored 0 were excluded, therefore the total scores ranged from 1 to 5.

#### Non-symbolic Addition

**FIGURE 1:**
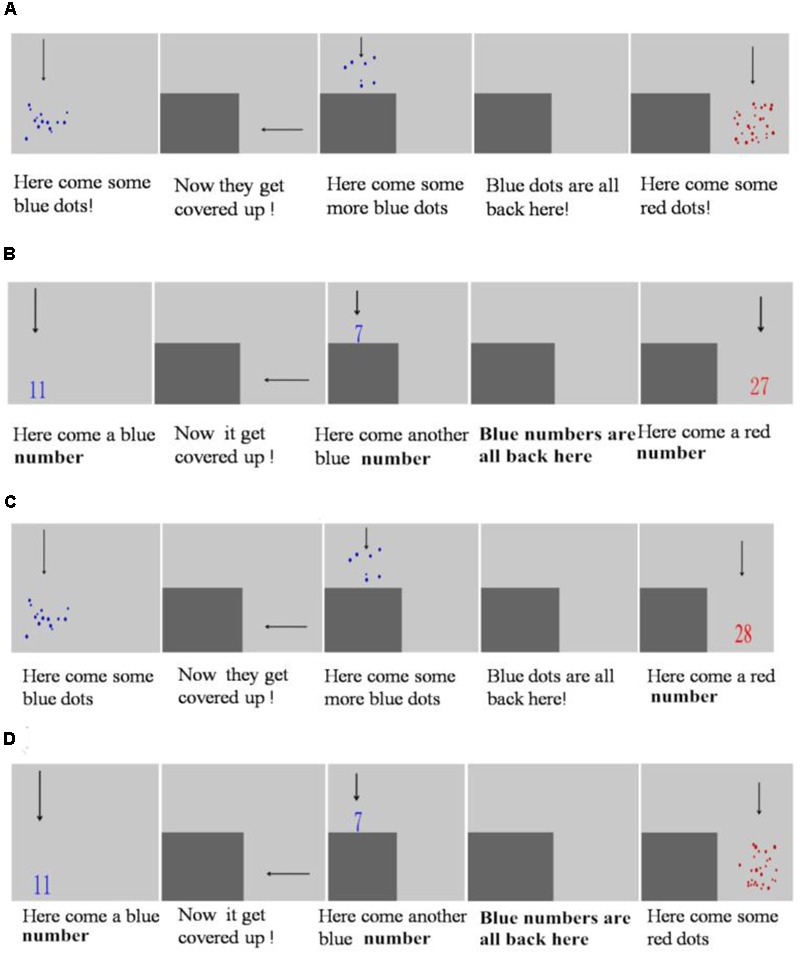
Schematic depictions of procedures and narrative for addition tasks. **(A)** An example trial of non-symbolic addition task. **(B)** An example trial of symbolic addition task. **(C)** An example trial of N-S addition version (comparing the sum of two arrays of dots with an Arabic number). **(D)** An example trial of S-N addition version (comparing the sum of two Arabic numbers with an array of dots). For each task, every animation event lasted 1400 ms, in other words, each trial lasted 7000 ms.

We devised a task (see **Figure 1A**) based on Barth et al.’s (2005) experimental paradigm. A trial entailed the following steps: (1) An array of blue dots moved onto the computer screen from the top, (2) These blue dots then were covered up by a gray box, (3) A second array of blue dots appeared above the gray box, (4) The second array of blue dots moved behind the gray box (i.e., All blue dots were all behind the gray box now), (5) Finally, an array of red dots appeared on the right side of the screen. Children were presented with these visual displays and the recorded verbal instructions simultaneously (Specific instructions were present in **Figure 1A**). In the end, they were asked “Which has more, the sum of blue dots or the set of red dots?” Participants pressed buttons to respond. They had a maximum of 10 s to respond and were required to respond as correctly and fast as possible. Each animated event lasted for 1400 ms, and the entire animation lasted for 7000 ms. All stimuli disappeared after the animation and the letters “F” and “J” were displayed on screen to remind children to press either button to respond. The inter-trial interval was 1000 ms. The visual displays were designed to be very short to prevent children from counting the dots. If children did not respond within the 10 s, the trial was automatically coded as incorrect. All children received three practice trials, followed by feedback (“√” or “×”) to make sure they understand the task. Then they received 24 test trials without feedback.

The numerosities included in this task ranged from 5 to 35. The ratios between the number of the blue dots and the number of red dots were 1/2 (easy), 2/3 (middle), and 3/4 (difficult). There were eight test trials at each ratio level. The comparisons were: 7+6 vs. 26, 9+7 vs. 8, 14+8 vs. 11, 11+5 vs. 32, 8+6 vs. 21, 7+5 vs. 8, 12+9 vs. 14, 11+7 vs. 27, 7+5 vs. 16, 9+7 vs. 12, 14+7 vs. 28, 15+9 vs. 18. Each comparison was repeated twice. The order of test trials was random. The dots were constructed in Microsoft Visual C++ 6.0, with size ranging from 0.2 to 0.6 cm. The physical features of the dots were controlled in respects to the total surface area and total contour length (Barth et al., 2006; Xenidou-Dervou et al., 2013, 2015).

#### Symbolic Addition

The task was identical to the non-symbolic addition task except that all dots were replaced by the corresponding Arabic numbers (see **Figure 1B**). Numbers used in all comparison sets were the same as those in the non-symbolic task. Researchers had demonstrated children were successful with verbal symbolic addition problems earlier than with written ones (Jordan et al., 2003). Therefore we did not include any verbal number words in the instruction. The recorded verbal instructions were identical to non-symbolic task, but instead of saying “some blue dots” and “some red dots,” we referred the stimuli as “a blue number” and “a red number.” Similarly, children were asked, “Which has more, the sum of the blue numbers or the red number?” Every animated event lasted for 1400 ms and then disappeared when participants were prompted to respond. All children received three practice trials and 24 test trials.

#### Hybrid Addition

Hybrid addition task was similar to the non-symbolic addition task and symbolic addition task except that each trial included both array of dots and Arabic numbers. Numbers used in all comparison sets were the same as those in the non-symbolic task. The design of these tasks was inspired by paradigms from Gilmore et al. (2007) and Mundy and Gilmore (2009). The task contained two versions: (1) The first two addends were arrays of dots and the comparison quantity was an Arabic number. This was similar to Mundy and Gilmore (2009) non-symbolic to symbolic mapping task (N-S task). In our N-S addition task, children would estimate the sum of two sets and convert the sum into a numeral, and then compare with another numeral. In other words, this was eventually converted into a numeral comparison (see **Figure 1C**). Children were asked, “Which has more, the sum of the blue dots or the red number?” (2) The first two addends were Arabic numbers, followed by an array of dots as the comparison quantity. This was similar to Mundy colleagues symbolic to non-symbolic mapping task (S-N task). In our S-N addition task, children would estimate the sum of two numerals and convert the sum into a set, and then compare with another set. The comparison was eventually converted into a set comparison (see **Figure 1D**). Children were asked, “Which has more, the sum of the blue numbers or the set red dots?” Similarly, every animation event lasted for 1400 ms and then the stimuli disappeared when participants were prompted to respond. For each task, children received three practice trials and 24 test trials.

### Procedure

Tasks used were computerized and presented in E-prime version 2.0 (Psychological Software Tools, Pittsburgh, PA, United States) with Lenovo Thinkpad E450. Children were individually tested in a quiet laboratory room, accompanied by one experimenter. A short break was provided in-between tasks. After the experiment, children received a small reward. All participants complete the number-naming task first and then the verbal-counting task. The experiment stopped for those with 0 score in the number-naming or verbal-counting task. In order to exclude the order effects, we used eight task sequences (see **Table 1** for details). Children were randomly assigned to one of the task sequences.

**Table 1:** Eight orders of tasks^1^.

Order 1	Order 2	Order 3	Order 4	Order 5	Order 6	Order7	Order 8
N	N	S	S	N-S	N-S	S-N	S-N
S	S	N	N	S-N	S-N	N-S	N-S
N-S	S-N	N-S	S-N	N	S	N	S
S-N	N-S	S-N	N-S	S	N	S	N

*Na, number-naming ability; VC, verbal-counting ability; N, non-symbolic addition task; S, symbolic addition task; N-S, non-symbolic to symbolic of hybrid addition task; S-N, non-symbolic to symbolic of hybrid addition task.*

*^1^We conducted a 4 (Task: non-symbolic, symbolic, N-S and S-N) × 3 (Ratio: 1:2, 2:3, 3:4) × 4 (Age: 4, 5, 6, 7 years old) × 8 (task order: 1, 2, 3, 4, 5, 6, 7, 8) repeated measures ANOVA on children’s performance accuracy. Results showed no significant main effect of task order, *F*(7,70) = 1.528, *p* = 0.172, and no significant interactions between order effect and other variables. Therefore, data collapsed across orders. In the subsequent analysis, task order was not considered as a factor.*

## Results

### Descriptive Statistics

**FIGURE 2:**
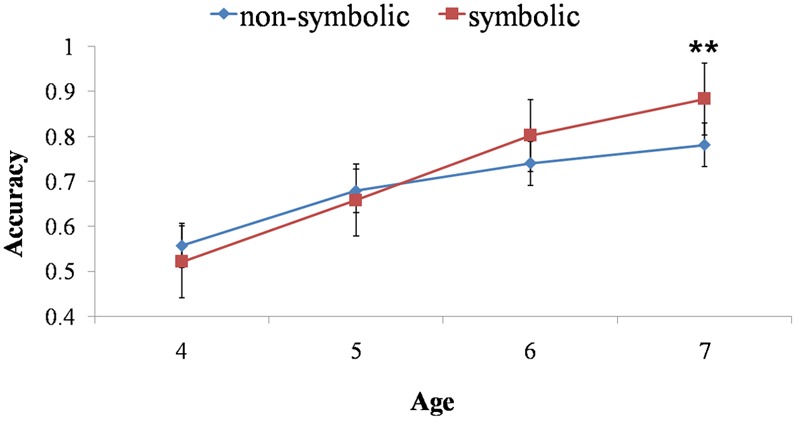
The interaction of age and task across non-symbolic and symbolic addition tasks. Children performed significantly better in symbolic addition task than non-symbolic one at 7 years old. The accuracy of symbolic and non-symbolic addition tasks had no difference for 4-, 5-, and 6-year-olds. ^∗∗^*p* < 0.01.

**FIGURE 3:**
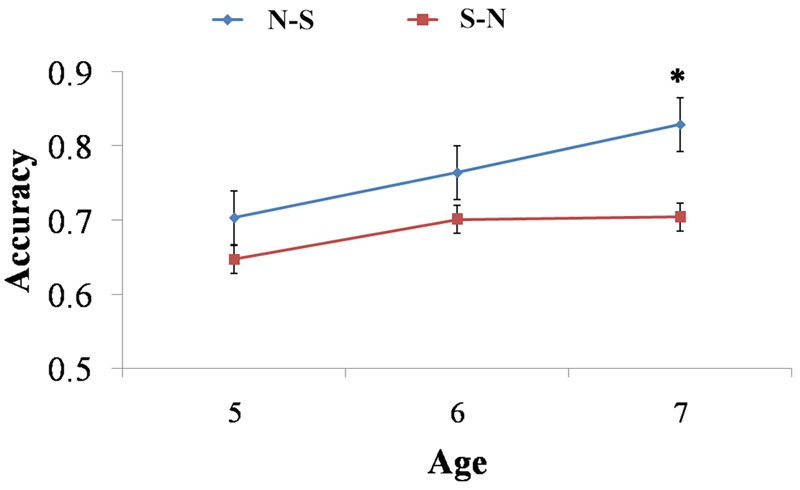
Children’s accuracy changed with age in N-S and S-N tasks. 7-year-olds performed significantly better in the N-S task. But there were no difference between the two tasks for 5- and 6-year-olds. ^∗^*p* < 0.05.

**Table 2** presented children’s scores for the number-naming task and the verbal-counting task, as well as their accuracy in four addition tasks. One-sample *t*-tests were conducted on accuracies in the non-symbolic, symbolic addition tasks and hybrid addition task (N-S and S-N tasks). The results showed all age groups performed well above chance-level in the non-symbolic addition task. However, in other three tasks, 5- to 7-year-olds performed above chance-level but not 4-year-olds. As hypothesized, children at 4 years old can complete non-symbolic addition. Children at 5 years old, before formal mathematical education, were able to do the symbolic addition task. Their performances on hybrid addition task indicated children can map between the two representations around 5 years old (see **Figures 2, 3**).

**Table 2:** Children’s performance in each numerical task.

	4 years old		5 years old		6 years old		7 years old	
	*M*	*SD*	*M*	*SD*	*M*	*SD*	*M*	*SD*
Na	1.610	0.629	2.600	0.498	3.000	0.000	3.000	0.000
VC	2.210	0.957	3.070	0.583	4.790	0.415	5.000	0.000
N	0.557^∗∗∗^	0.072	0.679^∗∗∗^	0.158	0.739^∗∗∗^	0.141	0.781^∗∗∗^	0.172
	d = 0.786		d = 0.635		d = 0.847		d = 0.734	
S	0.521	0.085	0.658^∗∗∗^	0.159	0.802^∗∗∗^	0.159	0.883^∗∗∗^	0.109
			d = 0.560		d = 0.729		d = 0.585	
N-S	0.517	0.111	0.703^∗∗∗^	0.180	0.763^∗∗∗^	0.198	0.829^∗∗∗^	0.211
			d = 0.615		d = 0.501		d = 0.694	
S-N	0.551	0.186	0.647^∗∗∗^	0.177	0.701^∗∗∗^	0.159	0.704^∗∗∗^	0.177
			d = 0.455		d = 0.620		d = 0.668	

*It shows children’s score in the numeracy task and the verbal-counting task, as well as their accuracy in the four addition tasks. We conducted one-sample *t*-tests on children’s accuracies in the four addition tasks (comparing with 50% chance level), ^∗∗∗^*p* < 0.001, d refers to the effect size. Na, number-naming ability; VC, verbal-counting ability; N, non-symbolic addition task; S, symbolic addition task; N-S, non-symbolic to symbolic of hybrid addition task; S-N, non-symbolic to symbolic of hybrid addition task.*

Correlation coefficients and Partial correlation coefficients (controlling for age) between different tasks are both presented in **Table 3**. Results showed there were strong associations between number-naming, verbal-counting skills and other four addition tasks. However, after controlling for age, the correlation coefficients between number-naming, verbal-counting abilities and other four addition tasks became insignificant. The result indicated age explains the associations among non-symbolic, symbolic addition and mapping skills. In other word, the number-naming and verbal-counting abilities had no significantly direct effect on non-symbolic, symbolic addition, and mapping skills. Both correlation and partial correlation showed strong associations among the four addition tasks. These results indicated the tasks were valid in capturing the underlying capacities and the non-symbolic, symbolic and mapping skills were closely linked with each other.

**Table 3:** Correlation coefficients and Partial correlation coefficients (controlling for age) between different numerical tasks.

		1	2	3	4	5
Na	r	1.000				
	r_p_	1.000				
VC	r	0.840^∗∗∗^				
	r_p_	0.612^∗∗∗^				
N	r	0.385^∗∗∗^	0.441^∗∗∗^			
	r_p_	0.027	0.019			
S	r	0.601^∗∗∗^	0.646^∗∗∗^	0.523^∗∗∗^		
	r_p_	0.165	0.096	0.264 ^∗∗^		
N-S	r	0.455^∗∗∗^	0.433^∗∗∗^	0.436^∗∗∗^	0.605^∗∗∗^	
	r_p_	0.119	-0.042	0.229^∗^	0.380^∗∗∗^	
S-N	r	0.290^∗∗^	0.275^∗∗^	0.335^∗∗^	0.430^∗∗∗^	0.378^∗∗∗^
	r_p_	0.080	-0.008	0.207^∗^	0.297^∗∗^	0.255^∗^

*It shows correlation coefficients and Partial correlation coefficients between different tasks. ^∗^*p* < 0.05, ^∗∗^*p* < 0.01, ^∗∗∗^*p* < 0.001. Na, number-naming ability; VC, verbal-counting ability; N, non-symbolic addition task; S, symbolic addition task; N-S, non-symbolic to symbolic of hybrid addition task; S-N, non-symbolic to symbolic of hybrid addition task.*

### The Development Trajectories of Non-symbolic and Symbolic Addition Ability

Regression analyses were carried out to examine the developmental trajectories of symbolic and non-symbolic addition capacities^1^. We conducted the slope difference test for the regression curves using Mplus7.0. The result suggested the developmental trajectories of the two capacities were significantly different, *t*(100) = 3.499, *p* < 0.001, *d* = 0.400. The result demonstrated different developmental trajectories of the non-symbolic and symbolic addition capacities (see **Figure 2**).

In order to provide detailed descriptions on the development of non-symbolic and symbolic addition capacities during childhood, we conducted a 2 (Task: non-symbolic and symbolic) × 3 (Ratio: 1:2, 2:3, 3:4) × 4 (Age: 4, 5, 6, 7 years old) repeated measures ANOVA on children’s performance accuracy. Mauchly’s test indicated that the assumption of sphericity had been violated for Ratio, χ^2^(2) = 10.760, *p* = 0.005. Therefore, we corrected the degrees of freedom by using the Greenhouse–Geisser estimates. The Box’s *M* test result for the homogeneity of variance hypothesis was significant (Box’s *M* test = 126.05, *F* = 3.760, *p* = 0.000). Results demonstrated the main effects of Ratio, *F*(1.810,196) = 19.677, *p* < 0.001, $\eta_{p}^{2}$ = 0.167, and Age, *F*(3,98) = 35.113, *p* < 0.001, $\eta_{p}^{2}$ = 0.518, and a significant interaction between Age and Ratio, *F*(6,196) = 3.348, *p* < 0.010, $\eta_{p}^{2}$ = 0.093. Further simple effect analyses and the Friedman non-parametric test indicated that, for 4-year-old children, there was no significant ratio effect, *F*(2,196) = 0.120, *p* = 0.889 [χ^2^(2) = 0.289, *p* = 0.866], and for other age groups, the ratio effect was significant, all *F*(2,196) > 3.17, *p* < 0.050, $\eta_{p}^{2}$ > 0.030 [all χ^2^(2) > 8.696, *p* < 0.013]. More importantly, our results also showed the significant interaction between Task and Age, *F*(3,98) = 3.833, *p* < 0.05, $\eta_{p}^{2}$ = 0.105. For this interaction, further simple effect analyses and the Wilcoxon non-parametric test demonstrated that, as expected, 7-year-olds were better at symbolic task than non-symbolic task, *F*(1,98) = 7.610, *p* < 0.010, $\eta_{p}^{2}$ = 0.071 (*Z* = -2.420, *p* = 0.016), but other age groups performed equally on the symbolic and the non-symbolic task, *F*_4-year-olds_(1,98) = 1.300, *p* = 0.256 (*Z* = -0.1.739, *p* = 0.082), *F*_5-year-olds_(1,98) = 0.480, *p* = 0.492 (*Z* = -0.795, *p* = 0.427), *F*_6-year-olds_(1,98) = 3.420, *p* = 0.067 (*Z* = -1.543, *p* = 0.123) (see **Figure 2**). The results demonstrated when children obtained formal mathematical education, their symbolic addition ability improved remarkably and it exceeded their non-symbolic ability in grade 1 (approximate age 7). There were no other significant interactions.

### Were Children Equally Proficient in the Two Mapping Directions?

Four-year-old children performed at chance level in hybrid addition tasks. Therefore, their data were eliminated from the following analysis. In order to illustrate children’s mapping abilities in both directions, we conducted a 2 (Task: N-S, S-N) × 3 (Ratio: 1:2, 2:3, 3:4) × 3 (Age: 5, 6, 7 years old) repeated measures ANOVA on their accuracy in the hybrid task. Mauchly’s test showed a violation of sphericity for the interaction between Task and Ratio, χ^2^(2) = 11.700, *p* = 0.003. Therefore, we corrected the degrees of freedom by using the Greenhouse–Geisser estimates. The Box’s *M* test result for the homogeneity of variance hypothesis was not significant (Box’s *M* test = 45.943, *F* = 0.955, *p* = 0.554). Results demonstrated the main effects of Ratio, *F*(1.956,142) = 58.856, *p* < 0.001, $\eta_{p}^{2}$ = 0.453, and Task, *F*(1,71) = 11.611, *p* < 0.010, $\eta_{p}^{2}$ = 0.141. Children performed significantly better in N-S task than S-N task (see **Table 2** and **Figure 3**). The interaction between Task and Ratio was also significant, *F*(1.733,142) = 6.597, *p* < 0.010, $\eta_{p}^{2}$ = 0.085. Further simple effect analyses showed at the ratio level 1:2, children’s performance did not differ between the two tasks, *F*(1,71) = 0.034, *p* = 0.563. But at the ratio level 2:3 and 3:4, their performance differed significantly between the two tasks, *F_2:3_*(1,71) = 4.320, *p* < 0.050, $\eta_{p}^{2}$ = 0.053, *F_3:4_*(1,71) = 17.710, *p* < 0.001, $\eta_{p}^{2}$ = 0.194. Children performed better in two versions of the hybrid addition task with the easy ratio, but have difficulties in S-N task with the difficult ratios. There were no other significant interactions.

*t*-Tests were conducted to further examine the age difference in mapping abilities in both directions. We compared the performance of 5-, 6-, and 7-year-old children in the N-S task and the S-N task. The results showed that 7-year-olds performed significant better on N-S task than they did on the S-N task, *t*(19) = 2.445, *p* = 0.024, *d* = 0.748, but there were no differences between the two tasks for 5-year-olds, *t*(29) = 1.691, *p* = 0.122, and 6-year-olds, *t*(23) = 1.605, *p* = 0.122 (see **Figure 3**). The results indicated children’s mapping from non-symbolic to symbolic representation became more accurate than the other direction in grade 1 (approximate age 7).

## Discussion

We investigated two issues in our study. First, we showed the detailed developmental trajectories of non-symbolic and symbolic addition skills during childhood. This is the first time that the issue has been systematically addressed for 4- to 7-year-olds. We found children were able to do non-symbolic addition at age 4 and they were able to do symbolic addition at age 5. Children’s accuracy of symbolic addition increased greatly after receiving formal school education, and it even exceeded the non-symbolic skills at 7 years old. Second, children’s mapping ability measured by our tasks suggested successful mapping between symbolic and non-symbolic representations of number from 5 years old. Specifically, 7-year-olds and older were more accurate in mapping non-symbolic representation to the symbolic one.

### The Developmental Trajectories of Non-symbolic and Symbolic Addition Abilities

Previous studies examined the non-symbolic addition ability in children as young as 5 years old (Barth et al., 2005, 2006, 2008; Xenidou-Dervou et al., 2015). There is no available evidence illustrating whether children younger than 5 would be able to do non-symbolic addition. Results from our study provided the first evidence showing 4-year-olds had developed the ability to do non-symbolic addition. The non-symbolic addition task in our study followed Barth et al.’s (2005) paradigm, with relatively easier ratios (1/2, 2/3, 3/4). Barth et al.’s (2005, 2006, 2008) and Xenidou-Dervou et al.’s (2015) studies utilized difficult ratios, such as 4/5, 4/6, 4/7. To some extent, this explains why children as young as 4 years old were able to do our non-symbolic addition task. Furthermore, why would ratio affect children’s performance on non-symbolic addition task? We think this is related to the approximate nature of the non-symbolic addition skill. The ANS has been claimed to support non-symbolic arithmetic (Barth et al., 2005, 2006, 2008), and it is assumed to be innately available (Wynn, 1992; Feigenson et al., 2004; Pica et al., 2004; Flombaum et al., 2005; Dehaene, 2011). When the task difficulty decreases, younger children are able to do non-symbolic addition.

Up to now, there is no consensus on when symbolic addition ability emerges (Gilmore et al., 2007; Xenidou-Dervou et al., 2015). Results from our study demonstrated that 5-year-olds performed above chance level on the symbolic addition task, which is in line with Gilmore et al.’s (2007) results. These results indicated children were equipped with symbolic addition capacity even before systematically learning the symbols (Gilmore et al., 2007). It is plausible that 5-year-olds solved the symbolic addition task with the help of ANS. However, Xenidou-Dervou et al. (2015) proposed that the ability of symbolic addition onsets at the 1st grade (approximately 6 years old), with the start of formal education. They found number-naming ability, which relates to language ability, was strongly correlated with the symbolic addition capacity, but not with non-symbolic one. However, in our study, we found, after controlling for age, the correlation coefficients between number-naming, verbal-counting abilities and non-symbolic, symbolic capacities were not significant, indicating non-significant relationships between the number-naming and verbal-counting abilities and the non-symbolic and symbolic addition skills. Our results differ from Xenidou-Dervou et al.’s (2015) because the number-naming test in our study was easier than that in Xenidou-Dervou et al. (2015). They used large numbers (such as 25, 36, 52, 21, 49, 67, 48, 24, and 63) to assessed children’s ability to name numbers above 20. Whereas test numbers in our task ranged from 5 to 32, with only five numbers above 20. Most 6 and 7 years old children could correctly name all numbers in our tasks (see **Table 2**). Moreover, our participants were Mandarin-speaking children, whereas Xenidou-Dervou et al.’s (2015) participants were Dutch-speaking children. Evidence from a variety of research areas indicates the superiority of early math achievement in Asian students, comparing to their Western counterparts (Suk-Han Ho and Fuson, 1998; Miller et al., 2000). This seems to be related to the regularity of number words in (East) Asian languages (Suk-Han Ho and Fuson, 1998; Miller et al., 2000). Asian languages, such as Mandarin, have a clear 10-structuralized number word system. It is claimed to lead to a better insight into numbers and superior arithmetic skills (Rasmussen et al., 2006; Van Luit and Van der Molen, 2011; Cankaya et al., 2014). Whereas Dutch language does not have a clear structure indicating 10 (Van Luit and Van der Molen, 2011). To some extent, these linguistic differences explain why Dutch-speaking children underperformed on numeracy tasks than their Mandarin-speaking peers (Van Luit and Van der Molen, 2011; Xenidou-Dervou et al., 2015).

Moreover, Chinese culture emphasizes more on math. Chinese parents always paid more attention to math (Siegler and Mu, 2008). They conduct more informal mathematics activities (most are symbolic tasks) with children than their Western counterparts. Chinese children are exposed to abundant quantitative information in daily life while playing number board game, discussing the amount of salt when cooking, discussing money when shopping, reading stories involved quantities and so on. Previous studies showed informal numeracy education predicted children’s early mathematics skills (Skwarchuk et al., 2014; Benavides-Varela et al., 2016). Therefore, the cultural emphasis on math in China and the rich exposure to mathematical knowledge may account for Chinese children’s better performance in symbolic addition tasks.

Our results provide detailed developmental trajectories of non-symbolic and symbolic arithmetic abilities during early childhood. We validated children’s symbolic addition ability improved largely with formal education and furthermore, their symbolic addition skill exceeded the non-symbolic one in grade 1 (approximate 7 years old). The different developmental trajectories of capacities in non-symbolic and symbolic addition may be attributed to the nature of non-symbolic and symbolic representations. Specifically, the non-symbolic addition capacity may depend on the ANS representation, which is considered inherent (McCrink and Wynn, 2004, 2009; Flombaum et al., 2005). Therefore, non-symbolic addition capacity is not directly affected by mathematical education. Whereas symbolic addition capacity was thought to be acquired (Pica et al., 2004; Xenidou-Dervou et al., 2015); children learn the symbolic number system when they obtain formal education. Their capacity in symbolic addition then improves progressively. We found at age 7 children’s symbolic addition skill exceeded non-symbolic one. In addition, the features of non-symbolic and symbolic representations also account for some of the differences in their developmental trajectories. Non-symbolic representation is approximate (Feigenson et al., 2004); whereas symbolic representation corresponds to precise quantities (Izard and Dehaene, 2008; Mussolin et al., 2014). Therefore, when the symbolic number system is taught explicitly in school, children’s performance on symbolic numerical tasks improves remarkably.

### When and How Children’s Mapping Ability Develops

We assessed children’s bi-direction mapping abilities using novel tasks and found that children from age 5 can map in both directions between symbolic and non-symbolic representations. Most previous research focused on such bi-direction mapping abilities in primary students (Mundy and Gilmore, 2009; Brankaer et al., 2014) and adults (Izard and Dehaene, 2008). Their results suggested a mature mapping ability in adults and even in primary students. Young children’s mapping ability was firstly investigated by Lipton and Spelke (2005). They found most 5-year-olds were not able to do the mapping tasks due to their less proficient counting skills. Most 5- to 6-year-old English-speaking children are able to count to 10, but only 47% of them can count to 20 (Muldoon et al., 2013). In contrast, most of our Mandarin-speaking 5- to 6-year-olds could verbally count to 20 or 30. This difference on counting skills may explain why 5-year-olds in our study can complete mapping tasks. Meanwhile, the abundant exposure to informal quantitative information in daily life may account for some of the different performance between Mandarin-speaking children and their counterparts in mapping task.

Regarding to which direction of mapping is dominant, previous studies (Mundy and Gilmore, 2009; Brankaer et al., 2014) showed different results. Mundy and Gilmore (2009) found children were more accurate on tasks involving mapping from non-symbolic to symbolic representation. However, no direction effect was found on mapping tasks by Brankaer et al. (2014). Results from our study suggested children were more accurate in mapping from non-symbolic to symbolic representation. In our N-S task, children estimated the sum of two sets of dots, converted the sum into a numeral, and then compared it with another numeral. Eventually they were comparing two numerals in a precise way. In our S-N task, children calculated the sum of two numerals, possibly converted the sum into a set of dots (for easy visual comparison), and then compared it with another set. Eventually, they were comparing two sets in an approximate way. Based on this speculation, we think children are more accurate on tasks involving mapping from non-symbolic to symbolic representation, which is in line with Mundy and Gilmore’s (2009) conclusion. Furthermore, we found this asymmetry of the bi-direction mapping abilities was significant for 7-year-olds. This is consistent with our results that 7-year-olds were more accurate in the symbolic addition than in the non-symbolic addition task. Children’s symbolic representation system is relatively mature at age 7.

### Limitations and Future Research

The current study has limitations and therefore requests future research to further clarify these questions. First, smaller numerosities such as 1–4, were not included in our tasks. Research (Feigenson et al., 2004) has shown that young children have developed a system to keep track of small numbers precisely. To prevent children from precise tracking dots in our addition tasks, we only considered numerosities larger than 4. However, the development trajectories of non-symbolic and symbolic addition skills and the mapping ability could be different for smaller numerosities from 1 to 4. Future research needs to address this issue and compare children’s non-symbolic and symbolic addition skills and mapping ability for large numerosities and small numerosities. Second, with the cross-sectional design of the current study, the developmental information provided by the data was limited. We were not able to examine longitudinally how non-symbolic and symbolic representations interact with each other. There is evidence showing children’s symbolic number knowledge affects their ANS system (Mussolin et al., 2014). Their cardinality proficiency and symbolic number knowledge predict later accuracy in numerosities comparisons. This requests future research to clarify the relationship between symbolic and non-symbolic representations of number. In fact, we are currently working on the follow-up of this study; with the longitudinal data, we would be able to draw a more comprehensive picture on the development of children’s numerical representation capacities.

Practically, future research also needs to investigate the relationship between children’s numerical representation capacities and their mathematic education and math performance. A large amount of research had shown the effect of numerical representation capacities on children’s mathematic performance (Schneider et al., 2009; Gobel et al., 2014; Friso-van Den Bos et al., 2015). On the other side, mathematical education also contributes to refine the phylogenetically inherited capacity to process large numbers approximately (Nys et al., 2013). It is worthwhile investigating the relation between numerical representation capacities and mathematics achievement. Future research in this direction will shed light on factors influencing children’s mathematical abilities and possible interventions to improve their mathematical performance.

## Author Contributions

YL contributed to conception and design, on acquisition and interpretation of data and on drafting of manuscript; MZ contributed to conception and design and drafting of manuscript; YC contributed to conception and design, and on interpretation of data; XZ contributed on interpretation of data; ZD contributed on interpretation of data; SY contributed to making the experimental materials.

## Conflict of Interest Statement

The authors declare that the research was conducted in the absence of any commercial or financial relationships that could be construed as a potential conflict of interest.

## Footnotes

1 Regression analyses were carried out separately for symbolic and non-symbolic addition abilities. We regarded age as the independent variable, because the number-naming and verbal-counting abilities were not significantly correlated with non-symbolic and symbolic addition tasks with controlling for age.
